# Trends in inpatient antibiotic use in Indonesia and the Philippines during the COVID-19 pandemic

**DOI:** 10.1017/ash.2025.48

**Published:** 2025-06-25

**Authors:** Amara Z. Fazal, Olivia L. McGovern, Garrett W. Mahon, Fernanda C. Lessa, Maria Tarcela Gler, Jemelyn Garcia, Mark John Festin, Kuntaman Kuntaman, Ida Parwati, Cherry Siregar, Jay Christian D. Muere, Gina De Guzman Betito, Maya Montemayor, Arleen De Leon, Emmeline Borillo, Mark Ramon Victor Llanes, Regina Berba, Musofa Rusli, Mariyatul Qibtiyah, Bambang Pujo Semedi, Rosantia Sarassari, Leonardus Widyatmoko, Basti Andriyoko, Adhi Kristianto Sugianli MD, Dewi Kartika Turbawaty, Ivo Dhanitri Ranita, Franciscus Ginting, Rahmadania Marita Joesoef, Made Ananda Krisna, Twisha S. Patel

**Affiliations:** 1 Centers for Disease Control and Prevention, Atlanta, GA, USA; 2 Makati Medical Center, Manila, Philippines; 3 Asian Hospital and Medical Center, Manila, Philippines; 4 Philippine General Hospital, University of the Philippines, Manila, Philippines; 5 Department of Clinical Microbiology, Dr. Soetomo General Academic Hospital, Surabaya, Indonesia; 6 Faculty of Medicine, Airlangga University, Surabaya, Indonesia; 7 Department of Clinical Pathology, Hasan Sadikin General Hospital, Faculty of Medicine, Padjadjaran University, Bandung, Indonesia; 8 Adam Malik Hospital, Medan, Indonesia; 9 Ejikman Institute for Molecular Biology, Jakarta, Indonesia; 10 Biology Department and Nuffield Department of Clinical Medicine, University of Oxford, Oxford, England

## Abstract

**Objective:**

Increased antibiotic use (AU) has been reported globally during the COVID-19 pandemic despite low rates of bacterial co-infection. We assessed changes in AU during the COVID-19 pandemic in Indonesia and the Philippines.

**Methods:**

We evaluated hospital-wide AU over 36 months in six hospitals, 3 in Indonesia and 3 in the Philippines. Intravenous antibiotics commonly used for respiratory conditions were selected and grouped for analysis. AU rates were calculated as monthly defined daily dose per 1000 patient-days or patient discharges. Median AU rates were compared from the pre-pandemic (March 2018–February 2020) and pandemic periods (March 2020–February 2021) using quantile regression to assess for statistical significance. Changes in AU during the COVID-19 pandemic were analyzed using interrupted time series analysis.

**Results:**

Significant increases were noted in the median AU rate from the pre-pandemic to pandemic period of all antibiotics combined in 3/6 hospitals (percentage change, Δ, 12.5%–63.6%) and anti-pseudomonal antibiotics in 3/6 hospitals (Δ 51.5%–161.5%). In the interrupted time series analysis, an immediate increase (range: 125.40–1762) in the use of all included antibiotics combined was observed in 3/6 hospitals at the onset of the COVID-19 pandemic. One of these 3 hospitals experienced a statistically significant sustained increase, while another experienced a decrease over time.

**Conclusions:**

We observed significant increases in facility-wide inpatient AU during the COVID-19 pandemic in our participating hospitals in Indonesia and the Philippines. These findings reinforce the importance of antibiotic stewardship practices to optimize AU, especially during infectious disease pandemics.

## Introduction

The morbidity and mortality of the coronavirus disease 2019 (COVID-19) pandemic is well documented with over 770 million cases and over 7 million deaths worldwide.^1^ A recognized consequence of the pandemic among hospitalized patients was increased antibiotic use (AU), with rates reported as high as 74.6%,^2–4^ despite relatively low rates of bacterial co-infection.^5^ This is a critical observation because antibiotic use is a known contributor to antimicrobial resistance (AMR),^6^ a leading global cause of death.^7,8^ Newer data demonstrate worsening AMR following the pandemic onset.^9–11^

Data on AU among hospitalized patients admitted during the COVID-19 pandemic from Southeast Asia are limited to a few countries,^8^ despite the World Health Organization (WHO) categorizing this region as one of the most affected by COVID-19 and the highest in the world for the risk of emergence and spread of AMR.^12^ A cross-sectional study of AU in patients hospitalized with SARS-CoV-2 infection at a tertiary hospital in the Philippines revealed that 57.3% received antibiotics, although only 1.9% had confirmed bacterial co-infection.^13^ A retrospective study of inpatients at a referral hospital in Indonesia found that patients with COVID-19 had a longer median duration of parenteral antibiotics as compared to patients without COVID-19.^14^ Although these studies provide some insight into AU trends during the COVID-19 pandemic, information remains limited on AU in these countries. Thus, we evaluated the effects of the COVID-19 pandemic on inpatient AU in Indonesia and the Philippines.

## Methods

### Study design and population

We conducted an ecological evaluation of hospital-wide AU in six hospitals, three in Indonesia and three in the Philippines. Data was collected retrospectively over a 36-month study period (March 2018–February 2021) and stratified into a 24-month pre-pandemic period (March 2018–February 2020) and a 12-month pandemic period (March 2020–February 2021). COVID-19 onset was defined as March 2020 as this is when the studied countries reported their first COVID-19 patient cases. Our study population included hospitalized adults (defined as ≥18 years of age) admitted to inpatient acute care wards. Non-acute care wards in which intravenous antibiotic use is typically low (ie, inpatient psychiatric units) or length-of-stay is low (ie, labor and delivery) were excluded. Data from patients in emergency departments were included if patients were boarded in that unit for >24 hours.

### Data collection

Hospitals were surveyed to assess various hospital characteristics and determine which antibiotics were used to treat respiratory and multidrug-resistant infections during the study period. AU data was collected from inpatient pharmacy dispensing records at all six hospitals and stratified by ward in which the antibiotic was dispensed. Data collection was limited to intravenously administered antibiotics to better assess trends among acutely ill inpatients. We included twenty-five individual intravenous antibiotics: imipenem, meropenem, piperacillin-tazobactam, cefotaxime, ceftriaxone, ceftazidime, cefepime, ceftaroline, ceftolozane-tazobactam, ceftazidime-avibactam, levofloxacin, moxifloxacin, ciprofloxacin, azithromycin, ampicillin-sulbactam, amoxicillin-clavulanate, polymyxin B, colistin, amikacin, gentamicin, aztreonam, vancomycin, linezolid, and tigecycline. Patient days were abstracted from hospital administrative records from five hospitals. One hospital (Hospital A) was unable to provide patient days, so patient discharge data was collected instead. AU rates were calculated for the 25 included antibiotics as monthly defined daily dose (DDD) per 1000 patient-days (or patient discharges for Hospital A).

### Statistical analysis

We first assessed hospital-wide changes in antibiotic use during the COVID-19 pandemic by comparing median AU rates in the 24-month pre-pandemic period to the 12-month pandemic period and calculating percent change. Quantile regression was then used to compare median AU rates and assess for statistical significance with a 95% confidence interval and a P-value of <0.05. This was done for four antibiotic categories: all 25 antibiotics combined; ceftriaxone; vancomycin and linezolid combined (antibiotics with activity against methicillin-resistant *Staphylococcus aureus* [MRSA], or anti-MRSA); and broad-spectrum antibiotics with activity against *Pseudomonas aeruginosa* (anti-PSA: imipenem, meropenem, piperacillin-tazobactam, ceftazidime, cefepime, ceftolozane-tazobactam, ceftazidime-avibactam, and levofloxacin). Of note, preliminary descriptive analyses of each antibiotic individually were performed initially, and final categories were made based on observed trends, prescribing practices at the included hospitals, and clinical relevance.

Additionally, we performed interrupted time series analyses with autoregressive models to assess the immediate change in AU with the onset of the COVID-19 pandemic and changes over time during the pandemic period. The following methodology is described in a parallel study conducted in South America.^15^ March 2020 was recorded as the time point to signify the COVID-19 pandemic onset period. The equation was defined as below:

*Y_t_* =*B*_0_+*B*_1_ *T*+*B*_2_ *X_t_* +*B*_3_(*T*−*T_i_*)*X_t_* +*E_t_*

where Y_*t*_ is the AU rate measured at time point *t*; *T,* the number of months from the start of the study period, ranging from 1 to 36; *X,* a dichotomous variable indicating the pandemic period (pre-pandemic, 0; pandemic, 1; and *T*_*i*_, the number of months that have transpired since the onset of the pandemic. The model parameter *B*_0_ represents the baseline intercept, *B*_1_, the pre-pandemic slope; *B*_2_, the change in step at the onset of the pandemic (immediate effect); *B*_3,_ the change in slope from pre-pandemic to pandemic; and *E,* the model’s error term.

A series of techniques were used to adjust for autocorrelation, seasonality, and secular trends.^16,17^ The Durbin-Watson test was used to detect autocorrelation, and when detected, the stepwise autoregressive process using the Yule-Walker method was performed for correction.^18^ When residual variance volatility was observed, a generalized autoregressive conditional heteroskedasticity approach was added, and the model with the lowest Akaike information criterion was selected.^19^ All statistical analyses were conducted using SAS (version 9.4), and an α value of <0.05 was considered statistically significant.

### Ethical approval

This activity was reviewed by CDC and was conducted consistent with applicable federal law and CDC policy^§^. The protocol was evaluated and approved by the institutional review board at each participating hospital: Adam Malik Hospital, Asian Hospital and Medical Center, Dr. Soetomo General Academic Hospital, Hasan Sadikin General Hospital, Makati Medical Center, and Philippine General Hospital.

^§^See e.g., 45 C.F.R. part 46.102(l)(2), 21 C.F.R. part 56; 42 U.S.C. §241(d); 5 U.S.C. §552a; 44 U.S.C. §3501 et seq.

## Results

### Facility characteristics

Of the 6 study hospitals, Hospitals A, B, C were in Indonesia and D, E, F were in the Philippines. Four were public hospitals (Hospitals A, B, C, and E), which served as referral centers for patients with COVID-19, and 2 were private hospitals (Hospitals D and F). All 4 of the public hospitals experienced increases in the number of ICU beds (range: 12%–98%) during the pandemic period. Five hospitals reported increases in the number of ventilators available for use (range: 8%–112%). Half of the facilities endorsed some staffing shortages during the pandemic, notably in microbiology lab technicians (Table 1). Hospital A noted delays in bacterial culture identification and antibiotic susceptibility testing during the pandemic period. All 6 hospitals had an antimicrobial stewardship program prior to the onset of the pandemic, with the most recent program established in 2019 (Hospital C). At least 3 hospitals reported changes within their stewardship programs during the pandemic, with decreased ability to review antibiotics as team members were redeployed to other roles and responsibilities (Table 1). Lastly, antibiotic availability was unaffected by the pandemic.

**Table 1.** Facility characteristics and reported changes during COVID-19 pandemic among hospitals in Indonesia and the Philippines (March 2020–February 2021)

Facility	Hospital A	Hospital B	Hospital C	Hospital D	Hospital E	Hospital F
**Country**	Indonesia	Indonesia	Indonesia	Philippines	Philippines	Philippines
**Private/Public**	Public	Public	Public	Private	Public	Private
**Referral center for COVID-19**	Yes	Yes	Yes	No	Yes	No
**Adult inpatient beds**	599	757	1471	295	560	265
**Change in ICU beds^a^**	*↑*98%	↑74%	↑4%	↓18%	↑12%	No change
**Change in ventilator availability^a^**	*↑*40%	↑112%	↑8%	↓6%	↑54%	↑48%
**Personnel shortages by specialty**	Microbiology lab technicians	None	None	ID physicians, nurses, infection preventionists, pharmacists	Microbiology lab technicians	None
**Empiric antibiotic use guidelines for patients with COVID-19 ^b^**	Levofloxacin and ceftriaxone	Azithromycin	For patients with suspected secondary bacterial pneumonia: levofloxacin, moxifloxacin, azithromycin, ceftriaxone, cefotaxime, vancomycin	Ceftriaxone and azithromycin for moderate pneumonia,piperacillin-tazobactam for severe pneumonia; empiric therapy discouraged in September 2020	None	Ceftriaxone and azithromycin
**Changes in antimicrobial stewardship practices**	Limited ability to review antibiotics	None	None	Restricted antibiotic requests moved to the EMR in 2020 for some wards; less oversight for restricted antibiotics	Approval process for levofloxacin, piperacillin-tazobactam, and ceftazidime discontinued	None
**Changes in microbiology laboratory practices**	Bacterial culture identification and AST delays due to staff diversion	None	None	None	None	None
**Changes to hospital**	None	Shift from paper to EMR	Shift from paper to EMR	None	Antibiotic ordering shifted to an electronic system	None

Abbreviations: AU, antibiotic use; AST, antibiotic susceptibility testing; ICU, intensive care unit; EMR, electronic medical record; ID, infectious disease.

a Percent change relative to before the pandemic.

b These guidelines varied throughout the pandemic without clear introduction dates and/or durations.

### Changes in antibiotic use

#### Changes in median antibiotic use

**Figure 1.**
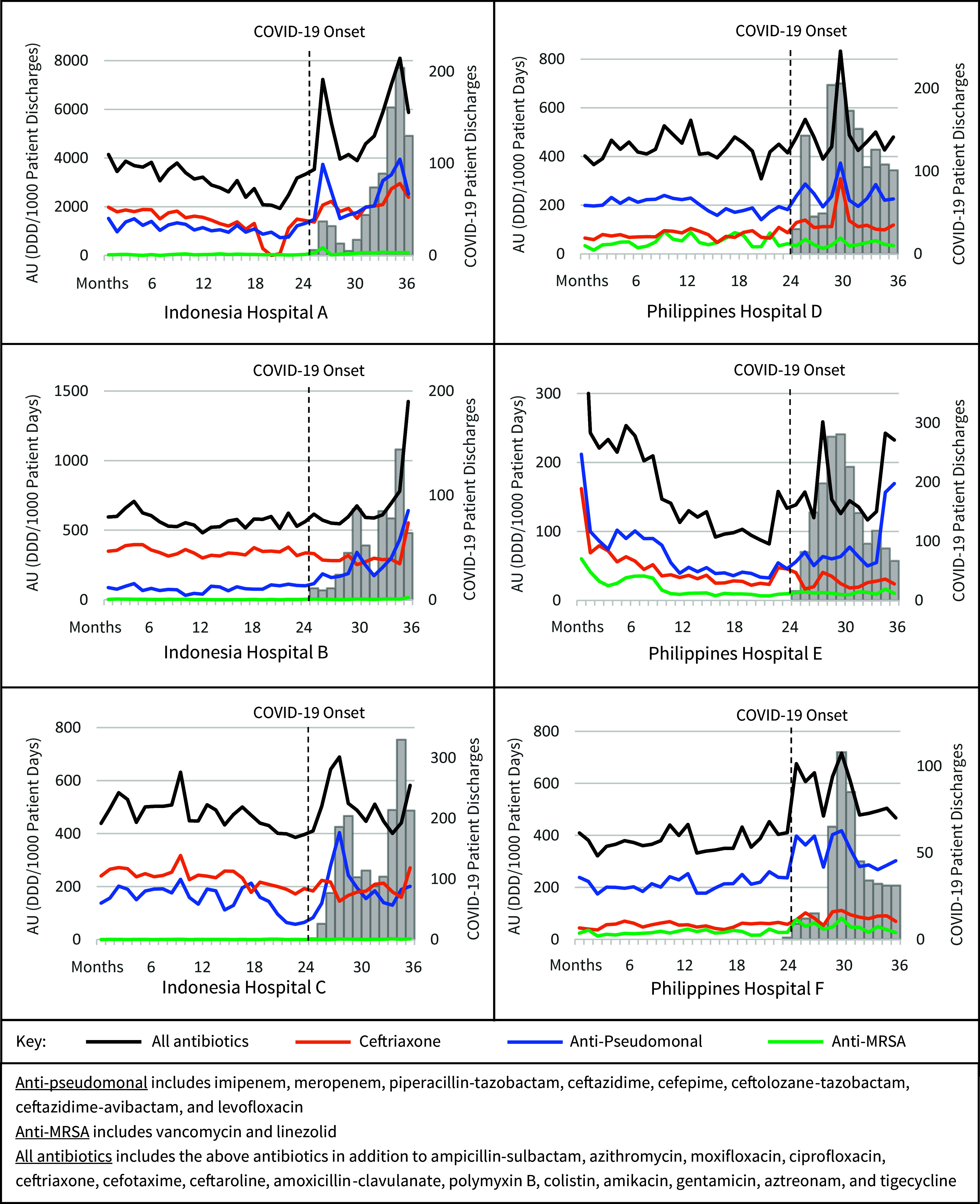
Intravenous antibiotic use (AU) and COVID-19 patient discharges in hospitals over time, March 2018–February 2021.

The median AU rate of all antibiotics combined increased significantly (*p* < 0.05) in 3 of the 6 hospitals (percentage change, Δ, 12.5%–63.6%; Hospitals A, D, F) during the COVID-19 pandemic period as compared to the pre-pandemic period (Table 2). All antibiotic use was higher during months with COVID-19 patient surges (Figure 1). In hospitals that experienced multiple surges of COVID-19 patients (Hospitals A, B, C, D), the increase in AU relative to the increase in COVID-19 cases was higher in the initial COVID-19 wave compared to subsequent waves.

**Table 2.** Median inpatient antibiotic use (DDD per 1000 patient days or discharges) among all included hospital wards in Indonesia (Hospitals A, B, C) and the Philippines (Hospitals D, E, F), March 2018–February 2021

		**Hospital A^f^**	Hospital B	Hospital C	Hospital D	Hospital E	Hospital F
**All Antibiotics ^a^**	Pre-pandemic^d^	3162.4	564.5	470.6	426.0	137.1	376.0
	Pandemic^e^	5172.5	603.4	495.7	479.0	141.5	554.6
	Percent change, Δ	63.6%	6.9%	5.3%	12.5%	3.2%	47.5%
	p-value	**0.031**	0.343	0.667	**0.023**	0.886	**0.004**
**Ceftriaxone**	Pre-pandemic^d^	1496.7	341.4	238.3	76.2	36.8	56.2
	Pandemic^e^	2051.6	287.0	183.0	115.2	25.1	87.3
	Percent change, Δ	37.1%	-15.9%	-23.2%	51.2%	-31.9%	55.4%
	p-value	**0.007**	**0.002**	**0.002**	**<0.001**	0.160	**<0.001**
**Anti-PSA ^b^**	Pre-pandemic^d^	1115.2	80.4	159.3	199.7	51.2	213.6
	Pandemic^e^	2277.3	210.2	186.7	231.9	63.4	323.8
	Percent change, Δ	104.2%	161.5%	17.2%	16.1%	23.8%	51.5%
	p-value	**0.035**	**0.016**	0.561	0.141	0.393	**0.005**
**Anti-MRSA ^c^**	Pre-pandemic^d^	34.4	2.5	0.4	46.8	10.4	25.9
	Pandemic^e^	107.4	4.0	0.9	37.9	11.0	47.2
	Percent change, Δ	212.6%	59.8%	111.8%	-19.0%	5.7%	82.6%
	p-value	**<0.001**	0.139	0.402	0.341	0.971	**0.005**

a All antibiotics include the below antibiotics in addition to ampicillin-sulbactam, azithromycin, moxifloxacin, ciprofloxacin, ceftriaxone, cefotaxime, ceftaroline, amoxicillin-clavulanate, Polymixin B, colistin, amikacin, gentamicin, aztreonam, and tigecycline.

b Anti-PSA (antibiotics with activity against *Pseudomonas aeruginosa*) includes imipenem, meropenem, piperacillin-tazobactam, ceftazidime, cefepime, ceftolozane-tazobactam, ceftazidime-avibactam, and levofloxacin.

c Anti-MRSA (antibiotics with activity against methicillin-resistant *Staphylococcus aureus*) includes vancomycin and linezolid.

d Pre-pandemic period: March 2018–February 2020.

e Pandemic period: March 2020–February 2021.

f DDD calculated per 1000 patient discharges as patient days data was not available.

During the pandemic period, ceftriaxone use increased significantly in 3 hospitals (Δ 37.1%–55.4%; Hospitals A, D, F), while in 2 hospitals it decreased significantly (Δ -15.9%– -23.2%; Hospital B and C respectively). Anti-pseudomonal antibiotic use increased significantly in three hospitals (Δ 51.5%–161.5%; Hospitals A, B, F). Although anti-MRSA AU rates were low overall, use increased significantly in two hospitals (Δ 82.6% and 212.6%, Hospital F and A, respectively).

Analysis of AU trends in critical care units revealed different patterns (Table S1 – supplementary material). Only one hospital’s ICUs demonstrated a statistically significant increase in overall AU from the pre-pandemic to pandemic period (Δ 33.5%, Hospital F).

#### Changes in antibiotic use at pandemic onset and trends over time

Three of the 6 hospitals (Hospitals A, C, F) experienced an immediate increase in the use of all included antibiotics combined at the onset of the COVID-19 pandemic (immediate effect estimate range, 125.40-1762.00) (Table 3). Despite the initial significant increase, Hospital F demonstrated a sustained significant decrease in AU rate over time during the pandemic period as compared to the pre-pandemic period (change in slope -23.37, *P* < 0.05) while Hospital A experienced a sustained increase in AU (change in slope +256.97). Two other hospitals also experienced a sustained increase in all antibiotic use over time during the pandemic period (Hospitals B and E; change in slope +34.16 and +13.93 respectively, *P* < 0.05).

**Table 3.** Parameter estimates from interrupted time series analysis of antibiotic use among hospitals in Indonesia (A, B, C) and the Philippines (D, E, F), March 2018–February 2021

		Immediate Effect of COVID-19 Pandemic		Impact of COVID-19 Pandemic Over Time			
		Estimate (SE)	p-value	Pre-Pandemic^d^ Estimate	Pandemic^e^ Estimate	Change in Slope (SE)	p-value
**Hospital A**	All Antibiotics^a^	1762.00 (552.65)	**0.003**	−66.66	190.31	256.97 (65.03)	**<0.001**
	Ceftriaxone	344.35 (355.99)	0.341	−44.56	113.47	158.03 (50.77)	**0.004**
	Anti-PSA^b^	957.63 (326.45)	**0.006**	−17.58	83.80	101.38 (38.45)	**0.013**
	Anti-MRSA^c^	131.25 (20.98)	**<0.001**	−0.16	−4.42	−4.26 (2.51)	0.091
**Hospital B**	All Antibiotics^a^	−62.86 (36.80)	0.088	−3.50	30.66	34.16 (3.44)	**<0.001**
	Ceftriaxone	−45.99 (9.82)	**<0.001**	−0.77	−1.00	−0.23 (1.18)	0.846
	Anti-PSA^b^	−12.04 (11.51)	0.296	3.27	28.69	25.42 (2.36)	**<0.001**
	Anti-MRSA^c^	0.10 (1.39)	0.942	−0.11	0.64	0.75 (0.17)	**<0.001**
**Hospital C**	All Antibiotics^a^	125.40 (47.26)	**0.012**	−4.70	−5.70	−1.00 (5.73)	0.862
	Ceftriaxone	−17.84 (19.57)	0.369	−3.56	2.34	5.90 (2.42)	**0.021**
	Anti-PSA^b^	62.09 (36.55)	0.100	−3.33	1.29	4.62 (4.54)	0.317
	Anti-MRSA^c^	−0.22 (0.23)	0.341	0.00	0.11	0.12 (0.03)	**<0.001**
**Hospital D**	All Antibiotics^a^	87.66 (102.34)	0.392	−0.14	−3.54	−3.40 (15.54)	0.827
	Ceftriaxone	67.53 (2.89)	**<0.001**	−0.27	−2.59	−2.32 (0.79)	**0.003**
	Anti-PSA^b^	77.42 (21.67)	**0.001**	−1.74	−1.15	0.59 (3.04)	0.985
	Anti-MRSA^c^	−21.80 (13.42)	0.114	0.96	−0.04	−1.01 (1.70)	0.558
**Hospital E**	All Antibiotics^a^	81.82 (43.49)	0.069	−9.95	3.98	13.93 (5.27)	**0.012**
	Ceftriaxone	15.65 (13.68)	0.261	−2.89	−0.42	2.47 (1.66)	0.147
	Anti-PSA^b^	14.37 (17.04)	0.399	−2.88	4.22	7.10 (2.44)	**0.004**
	Anti-MRSA^c^	1.72 (62.90)	0.978	−1.94	0.44	2.38 (4.21)	0.572
**Hospital F**	All Antibiotics^a^	337.37 (8.28)	**<0.001**	0.11	−23.26	−23.37 (1.17)	**<0.001**
	Ceftriaxone	29.15 (9.08)	**0.003**	0.37	−0.21	−0.58 (1.10)	0.601
	Anti-PSA^b^	162.68 (11.78)	**<0.001**	2.16	−12.59	−14.75 (1.38)	**<0.001**
	Anti-MRSA^c^	39.25 (7.31)	**<0.001**	0.28	−3.06	−3.34 (0.89)	**<0.001**

a All antibiotics include the below antibiotics in addition to ampicillin-sulbactam, azithromycin, levofloxacin, moxifloxacin, ciprofloxacin, ceftriaxone, cefotaxime, ceftaroline, amoxicillin-clavulanate, Polymixin B, colistin, amikacin, gentamicin, aztreonam, and tigecycline.

b Anti-PSA (antibiotics with activity against *Pseudomonas aeruginosa*) includes imipenem, meropenem, piperacillin-tazobactam, ceftazidime, cefepime, ceftolozane-tazobactam, ceftazidime-avibactam, and levofloxacin.

c Anti-MRSA (antibiotics with activity against methicillin-resistant *Staphylococcus aureus*) includes vancomycin and linezolid.

d Pre-pandemic period: March 2018–February 2020.

e Pandemic period: March 2020–February 2021.

Ceftriaxone use increased at Hospitals D and F immediately at the onset of the pandemic period (immediate effect estimate 67.53 and 29.15 respectively, *P* < 0.05), then decreased over time in Hospital D (change in slope -2.32, *P* < 0.05). Ceftriaxone use also decreased significantly over time in Hospital C, where there was no statistically significant immediate effect at the onset of the pandemic. Hospital B experienced a statistically significant decrease in ceftriaxone use at the start of the pandemic period (immediate effect estimate -45.99, *P* < 0.05). While Hospital A did not demonstrate an immediate increase in ceftriaxone use at the onset of the pandemic, it did experience increased use over time during the pandemic (change in slope +158.03, *P* < 0.05).

Anti-pseudomonal antibiotic use increased at 3 hospitals immediately at the onset of the pandemic (Hospitals A, D, F; immediate effect estimate range 77.42-957.63, *P* < 0.05) with a sustained increase during the pandemic period at only Hospital A (change in slope +101.38, p<0.05) and subsequent decrease at Hospital F (change in slope -14.75, *P* < 0.05). Two other hospitals (Hospital B, E) had no immediate change in anti-pseudomonal AU at the onset of the pandemic but experienced a significant increase in use during the pandemic period (changes in slope +25.42 and +7.10 respectively, *P* < 0.05).

Anti-MRSA antibiotic use increased at 2 hospitals (Hospitals A, F) immediately at the onset of the pandemic (immediate effect estimate 131.25 and 39.25 respectively, *P* < 0.05). In Hospital F, this use subsequently decreased during the pandemic period (change in slope -3.34, *P* < 0.05). While Hospitals B and C saw no immediate increase in anti-MRSA use at the onset of the pandemic, both experienced slight increases during the pandemic period (change in slope +0.75 and +0.11, respectively, *P* < 0.05).

## Discussion

We observed significant increases in facility-wide inpatient AU during the COVID-19 pandemic in our participating hospitals in Indonesia and the Philippines, similar to reports from other regions worldwide.^15,20^ Of note, increases in ceftriaxone (median AU Δ 37.1%–55.4%) and anti-pseudomonal antibiotics (median AU Δ 51.5%–161.5%) were observed, which is concerning as overuse of these antibiotics is a known contributor to antibiotic resistance among gram-negative bacteria. Furthermore, AU in our study hospitals appeared to rise dramatically during early COVID-19 patient surges, as found in South America, suggestive of increased use during periods of high COVID-19 burden (Figure 1). Two of the 3 hospitals in Indonesia (Hospital A and C) experienced an immediate increase in ≥1 antibiotic group according to the interrupted time series analysis. A similar result was found in the Philippines, whereby two hospitals (D and F) experienced an immediate increase in ≥1 antibiotic group. Diagnostic uncertainty, lack of alternative therapies, early national guidelines recommendations, and concern for bacterial co-infection were reported to be among the top reasons for early AU by site investigators.

Notably, AU trends varied across our study hospitals, likely reflecting the diversity in healthcare systems, public and private hospital funding, and patient populations. For example, of the two hospitals (Hospital A and F) in the region that experienced a significant increase in median AU for all 4 antibiotic groups, one (Hospital A in Indonesia) experienced sustained increases over time among all AU while the other (Hospital F in the Philippines) experienced an initial increase followed by a significant decrease in AU rate over time among all AU. These results show that high rates of early AU were not always sustained. Hospital F had a mechanism in place to monitor AU and implemented measures, including targeting the overuse of antibiotics. Although decreases in AU in Hospital F were observed after measures were implemented, it is important to note that rates of AU, including broad-spectrum anti-pseudomonal AU, were still higher at the end of our study period than during the pre-pandemic period. This suggests further improvements could be made. Of the two hospitals (Hospital B and C) that experienced statistically significant decreases in ceftriaxone, one (Hospital B) found this decrease in ceftriaxone use to be paired with a significant increase in anti-pseudomonal AU, suggesting a shift towards the use of broader spectrum agents. This striking trend was not found in Hospital C, perhaps reflecting a more successful antibiotic stewardship initiative to reduce unnecessary antibiotic use during the pandemic.

Further, as seen in our South American study, we did not find that median AU rates had statistically significant increases in ICUs as compared to acute care wards from the pre-pandemic to pandemic period. Only one hospital experienced significant increases in overall AU in the ICUs (Hospital F). These findings demonstrate that AU in ICUs did not drive overall increases seen in AU during the pandemic. Although four of the six hospitals experienced increases in the number of ICU beds, suggesting an expanded volume of critically ill patients, the burden of COVID-19 impacted the entire facility.

Lastly, one surprising observation was the overall low usage of anti-MRSA agents in our study hospitals, which stands in contrast to the known high rates of MRSA in Indonesia and the Philippines. Hospital staff in both countries endorsed the costly nature of vancomycin as a barrier to prescription, in addition to strict antibiotic stewardship policies at some hospitals, which require positive bacterial culture prior to prescription. Of note, while we observed some increases in anti-MRSA antibiotic use in two of our study hospitals in the Philippines, MRSA rates in the Philippines have been declining steadily over the past several years, from 53% in 2018 to 40.5% in 2023.^21,22^

Several of the study hospitals provided antibiotic use guidelines for patients with suspected or diagnosed COVID-19 (Table 1), and some recommended regimens included ceftriaxone (Hospitals D, F), while others recommended levofloxacin (Hospitals A, B). Unsurprisingly, we observed statistically significant increases in these respective antibiotic classes for these hospitals during the pandemic, even though some recommendations for AU in patients with COVID-19 were redacted in late 2020. Notably, in some hospitals with multiple waves of COVID-19 patients, there appeared to be proportionally less AU in subsequent waves as compared to the first wave (Hospital A, C, D) (Figure 1). This may be due to hospital-wide strengthening of AU practices as reported by the study hospitals and other successful antibiotic stewardship practices.

All study hospitals reported having active antimicrobial stewardship (AMS) programs prior to the pandemic, and at least 3 hospitals reported that their AMS programs were affected by the pandemic as team members were redeployed to other roles and responsibilities. Changes in AMS programs have been noted in other regions of the world to varying degrees during the COVID-19 pandemic.^23^ Several considerations should be noted that may have introduced vulnerabilities to the study hospitals’ AMS programs. Discussions with hospital staff revealed half of the study hospitals did not have any full-time AMS program staff, with staff dedicating 20-50% of their time to performing antibiotic stewardship actions. While all AMS programs were multidisciplinary, they were composed largely of physicians (20%–90% of each team’s composition) with more limited involvement of pharmacists (10%–40%). Resiliency of these programs may be improved with staff who are fully dedicated to antibiotic stewardship activities in addition to greater support for the training and involvement of clinical pharmacists.^24^ Remarkably, many of our study hospitals have augmented antibiotic stewardship efforts since the onset of the pandemic with adaptations of WHO AWaRe guidelines^25^ and stricter antibiotic preauthorization requirements. This heightened awareness of the importance of AMS programs can be considered one of the silver linings of the COVID-19 pandemic, which exposed areas of the healthcare system in special need of reinforcement.^8^

Our study has important limitations to consider. Antibiotic use data was collected at the hospital ward level, so we are unable to stratify analyses by patient-level data such as COVID-19 diagnosis, presence of bacterial infection, length of stay, or disease severity. Thus, antibiotic use rates are indicative of overall facility-level trends and cannot be attributed directly to patients hospitalized with COVID-19. Data collection was limited to intravenously administered antibiotics to facilitate accurate data collection and better assess trends among inpatients, so results cannot be generalized to outpatients and do not account for inpatients taking oral antibiotics. As our study population was limited to adults (defined as ≥18 years of age), results may not be generalizable to pediatric populations.

In conclusion, we observed significant increases in intravenous antibiotic use in hospitals in Indonesia and the Philippines during the COVID-19 pandemic. The increased use of broad-spectrum antibiotics is of particular concern given observations of worsening AMR worldwide and in the high-risk Southeast Asia region. While our study adds important new information about patterns of AU in these countries, especially under periods of strain on the healthcare system, more research on AU in the region is needed to better understand the differences we observed. Further research on the impact of the increased AU on AMR and the effectiveness of antibiotic stewardship interventions is needed to strengthen the response to future pandemics in Southeast Asia. Our results highlight the need to bolster AMS programs to guide appropriate antibiotic use, both at baseline and as an early pandemic preparedness effort. Further, AMS programs should work collaboratively with other hospital emergency response committees to ensure these actions are sustained throughout a pandemic.

## Supporting information

10.1017/ash.2025.48.sm001 Fazal et al. supplementary material Fazal et al. supplementary material

## Supplementary material

The supplementary material for this article can be found at https://doi.org/10.1017/ash.2025.48
