# Supplementary material for: Trends in inpatient antibiotic use in Indonesia and the Philippines during the COVID-19 pandemic
Source: Antimicrob Steward Healthc Epidemiol. 2025 Jun 25;5(1):e134. doi: 10.1017/ash.2025.48 (PMC12188270; doi:10.1017/ash.2025.48)
Supplement: Fazal et al. supplementary material [file S2732494X25000488sup001.pdf]

Appendix

Table 1. Median inpatient antibiotic use (DDD per 1000 patient days or discharges) among hospital intensive care units in Indonesia (Hospitals A, B, C) and the Philippines (Hospitals D, E, F), March 2018 – February 2021

		Hospital A[f]	Hospital B	Hospital C	Hospital D	Hospital E	Hospital F
**All Antibiotics[a]**	Pre-pandemic[d]	77903.36	1133.08	823.95	724.83	621.76	771.52
	Pandemic[e]	73014.88	1052.81	774.90	99.265	787.38	1030.26
	Percent change, Δ	-6.3%	-7.1%	-6.0%	36.9%	26.6%	33.5%
	p-value	0.911	0.967	0.832	0.066	0.263	**0.003**
**Ceftriaxone**	Pre-pandemic[d]	15209.82	443.82	323.75	55.02	56.82	44.51
	Pandemic[e]	14788.89	290.18	105.22	71.65	37.84	18.81
	Percent change, Δ	-2.8%	-34.6%	-67.5%	30.2%	-33.4%	-57.5%
	p-value	0.989	0.106	**<0.001**	0.473	0.293	0.063
**Anti-PSA[b]**	Pre-pandemic[d]	47238.10	334.22	310.26	436.67	317.04	516.23
	Pandemic[e]	43913.10	290.56	421.10	641.08	450.75	659.07
	Percent change, Δ	-7.0%	16.9%	35.7%	46.8%	42.4%	27.7%
	p-value	0.987	0.942	0.234	**0.049**	0.205	0.099
**Anti-MRSA[c]**	Pre-pandemic[d]	1135.71	10.38	0.00	79.74	70.76	89.74
	Pandemic[e]	3569.44	0.00	9.84	156.81	85.46	163.90
	Percent change, Δ	214.3%	-100.0%	N/A	96.7%	20.8%	82.6%
	p-value	0.086	0.411	0.876	**0.031**	0.884	**0.008**

[a]All antibiotics includes the below antibiotics in addition to ampicillin-sulbactam, azithromycin, moxifloxacin, ciprofloxacin, ceftriaxone, cefotaxime, ceftaroline, amoxicillin-clavulanate, Polymixin B, colistin, amikacin, gentamicin, aztreonam, and tigecycline

[b]Anti-PSA (antibiotics with activity against *Pseudomonas aeruginosa*) includes imipenem, meropenem, piperacillin-tazobactam, ceftazidime, cefepime, ceftolozane-tazobactam, ceftazidime-avibactam, and levofloxacin

[c]Anti-MRSA (antibiotics with activity against methicillin-resistant *Staphylococcus aureus*) includes vancomycin and linezolid

[d]Pre-pandemic period: March 2018 – February 2020

[e]Pandemic period: March 2020 – February 2021

[f]DDD calculated per 1000 patient discharges as patient days data was not available
